# Hindrances to Medical Recruiting

**Published:** 1915-08-21

**Authors:** 


### The Hospital

The Workers' Newspaper of Administrative Medicine and Institutional Life, Administration, National Insurance and Health.

No. 1523, Vol. LVIII. SATURDAY, AUGUST 21, 1915. PRICE ONE PENNY

### HINDRANCES TO MEDICAL RECRUITING.

It is still necessary to proclaim that the Army is in urgent need of a more abundant supply of doctors. The terms of the most recent statement made by Sir Alfred Keogh are substantially identical with those of the invitation he issued earlier in the year. The claims of his Department, he tells us, cannot be put too strongly, and he wants all the medical officers he can possibly get. In plain terms, every medical man of military age is told that it is his duty to offer himself for service, and that if he fails to do so he must be prepared to justify his refusal both to his own conscience and to the nation of which he forms a part. No more serious responsibility could be presented to the profession, and the occasion must be met beyond risk of challenge.

It is unfortunate that the demand presented in this fashion cannot be accompanied by the guarantee of facts and figures. Medical practitioners, perhaps to a larger extent than any other class of the community, are occupied in work in which decision and judgment rest on evidence, and therefore they, in an exceptional degree, feel the need for information as an essential preliminary to action. Appeals which are general and more or less vague appear to them to be wanting in support, and are thus apt to be postponed until the evidence necessary to justify them is produced. In the present position this evidence, we are told, cannot be produced. Military considerations forbid such a step as one capable of giving to the enemy information which might be prejudicial to the welfare and success of our fighting forces. Personal decision must, therefore, be taken without the opportunity thoroughly to explore the position. Yet it cannot be doubted that there is an urgent patriotic claim for the acceptance of the judgment of the military medical authorities. Short of an actual statement of the strength of the new Armies and of the number of medical practitioners required by these, no stronger terms could be employed than those selected by Sir Alfred Keogh and endorsed, on the strength of confidential information communicated to them, by the recently appointed War Emergency Committee. This Committee may fairly claim to be representative of the profession, and it speaks seriously and even solemnly of the urgency of the situation. It is, therefore, a fair thing to say that the actual facts have been submitted to those in whom the profession ought to have full confidence, and that these facts justify the demands which the War Office has advanced. No man, therefore, can possibly be justified in a refusal to respond to these demands either on the irresponsible rumour that the authorities have an abundant supply of doctors, or because no official figures announce exactly how many practitioners are judged to be essential. Silence in both of these directions is vital to military success. Therefore the medical profession, like the rest of the nation, must be content to trust the judgment of those charged with the direction of national affairs. Every fit man of military age is wanted: consequently it is the duty of each fit man of military age to volunteer. To hesitate because the details of the situation are not disclosed can only be condemned as egotistical, selfish, and unpatriotic; it is to prove oneself unequal to the greatness of the occasion and deaf and blind to the acuteness of the emergency.

In addition to the necessary secrecy of the position, a second influence which has opposed the success of medical recruiting is the circulation of reports which suggest that many of those who have already joined the R.A.M.C. find themselves with but scanty occupation. They are kept at routine duties which leave much time on their hands and which appear to have but a remote relation to the success of the war. Even men who have been sent to France tell us that the service seems to them to be overstaffed and that they very often have little or nothing to do. These complaints surely argue some failure to appreciate the true nature of the position. Army service is in certain respects different from civil practice, and it cannot be successfully undertaken without some measure of preparation. Anxiety to get to the Front is very excellent and very natural, but it is necessary to be ready to be serviceable on arrival, and this means time spent in routine duties prior to departure. Of still greater importance in relation to the point here at issue is the consideration that the authorities must be ready for extensive and often sudden developments. It is the maximum demand for which provision has to be made, and were anything less than this proposed, there would be shortage and failure, and therefore suffering and loss, at the very time when help was most needed. Hence it is inevitable that there must be times, even in the fighting area, when doctors, like their combatant comrades, seem to be in excess, and when individuals appear to lack a justification for their official existence. Here, again, the guide must be the decision of the authorities and not the judgment of the individual. This decision announces plainly that more doctors are urgently needed, and whoever challenges it must be dismissed as a person who entertains an unduly exalted value of the worth of his own opinion.

There remains the question of money. For many to whom the call of duty comes, a response means, undoubtedly, the risk of financial loss. Little can be said in relief other than that the time is one for sacrifice, and that the need for this falls upon doctors as upon other members of the community. Individuals must be content to trust their interests to the loyalty of their professional brethren who remain at home. As we have said before, we should like to see some fund raised in the profession generally, both as an evidence of the corporate recognition of the nation's claim and as a source from which compensation to individuals could be effected. But whether this is or is not possible, the claim is clear and urgent, and we cannot think it will fall on deaf ears.

